# Isolated Flailed P3 Scallop of the Mitral Valve Leaflet in the Setting of Newly Diagnosed Heart Failure With Preserved Ejection Fraction

**DOI:** 10.7759/cureus.9919

**Published:** 2020-08-21

**Authors:** Jesus Salas Noain, Eddy Mizrahi, Shengnan Zheng, Arun Minupuri

**Affiliations:** 1 Internal Medicine, Mercy Catholic Medical Center, Darby, USA; 2 Cardiology, Mercy Philadelphia Hospital, Philadelphia, USA

**Keywords:** mitral valve prolapse, mitral valve insufficiency, congestive heart failure, multi-vessel disease

## Abstract

Mitral valve prolapse (MVP) is characterized by typical fibromyxomatous changes in the mitral leaflet tissue with superior displacement of one or both leaflets into the left atrium. An echocardiogram is a fundamental study required for the diagnosis of MVP with a flail leaflet and grading of mitral regurgitation (MR) severity. Most patients with MVP have a risk of cardiovascular morbidity and mortality similar to that of the general population, though moderate to severe MR and left ventricular (LV) ejection fraction less than 50% have been postulated to increase the risk of adverse cardiac events. In this case report, we present an isolated flailed P3 scallop of the mitral valve leaflet leading to severe MR and acute congestive heart failure.

A 54-year-old African-American male with a medical history of hypertension, hyperlipidemia, and transient ischemic attack, presented to the emergency department (ED) for evaluation of dyspnea on exertion. The patient reported that his dyspnea started one week prior to ED visit and was associated with intermittent chest pain. He also endorsed mild orthopnea and lightheadedness, though he denied any syncopal event. Vital signs were found within normal limits on arrival. He clinically appeared to be volume overloaded which improved quickly with IV furosemide. Transesophageal echocardiogram (TEE) with 3D image acquisition showed significant for hyper-dynamic LV function and evidence of isolated flailed P3 scallop of the mitral valve (MV) leaflet resulting in a severe eccentric, anteriorly directed MR jet. The MV leaflets did not appear thickened, and there was no evidence of mitral or aortic stenosis. Cardiac catheterization showed multivessel disease for which the patient underwent coronary artery bypass grafting and MV repair.

This patient presented with new-onset congestive heart failure secondary to severe MR associated with undiagnosed MVP. Commonly, the middle scallop (P2) of the posterior leaflet is more prone to prolapse due to its redundancy and variable thickness with the impact of greater systolic pressure. However, in this case of acute severe MR, we identified an isolated flail of the P3 segment. We believe that this rare TEE finding was associated with a torn chordae or ruptured papillary muscle secondary to ischemic disease as the posteromedial papillary muscle has a single blood supply and is particularly prompted to injury from myocardial infarction.

## Introduction

Mitral valve prolapse (MVP) is characterized by typical fibromyxomatous changes in the mitral leaflet tissue with superior displacement of one or both leaflets into the left atrium [1]. An echocardiogram is a fundamental study required for the diagnosis of MVP with a flail leaflet and grading of mitral regurgitation (MR) severity [2]. A systolic billowing of any portion of the mitral leaflets of 2 mm or more above the annular plane in a long-axis view on echocardiogram is diagnostic for MVP [3]. MVP with a flail leaflet is a common cause of acute or sub-acute severe MR [4].

MVP is present in approximately 2% to 3% of the general population, with a slightly higher prevalence in women than men. It is a common cause of mitral regurgitation; however, most patients with MVP have only trace to mild MR, with severe MR noted in less than 5% of the patients at initial diagnosis [5]. MVP is associated with multiple complications including transient ischemic attack or stroke, cardiac arrhythmias (atrial or ventricular premature beats and ventricular or supraventricular tachycardia), worsening MR, and congestive heart failure, and infective endocarditis [6]. Most patients with MVP have a risk of cardiovascular morbidity and mortality similar to that of the general population, though moderate to severe MR and left ventricular ejection fraction less than 50% have been postulated to increase the risk of adverse cardiac events. Other risk factors for cardiovascular morbidity include flail mitral leaflet, left atrium size greater than 40 mm, atrial fibrillation, and age above 50 years [7,8]. 

Carpentier’s widely recognized nomenclature describes three posterior leaflet scallops, the lateral (P1), middle (P2), and medial (P3) - and three anterior segments - lateral (A1), middle (A2), and medial (A3) [9]. Segments A1, P1, and PM1 attach to the anterolateral papillary muscle, and segments A2, P2, and PM2 to the posteromedial papillary muscle. A billowing motion of the posterior leaflet may occur with pathological and redundant tissue and is considered abnormal [9]. The abnormal billowing of the anterior and/or posterior leaflets is often associated with myxomatous thickening and excess tissue. The majority of MVP cases involves the posterior middle scallop. The most common segment involved in MVP is the P2 segment [10].

In this case report, we present a very atypical case of isolated flailed P3 scallop of the mitral valve leaflet associated with acute severe MR. 

## Case presentation

**Figure 1:**
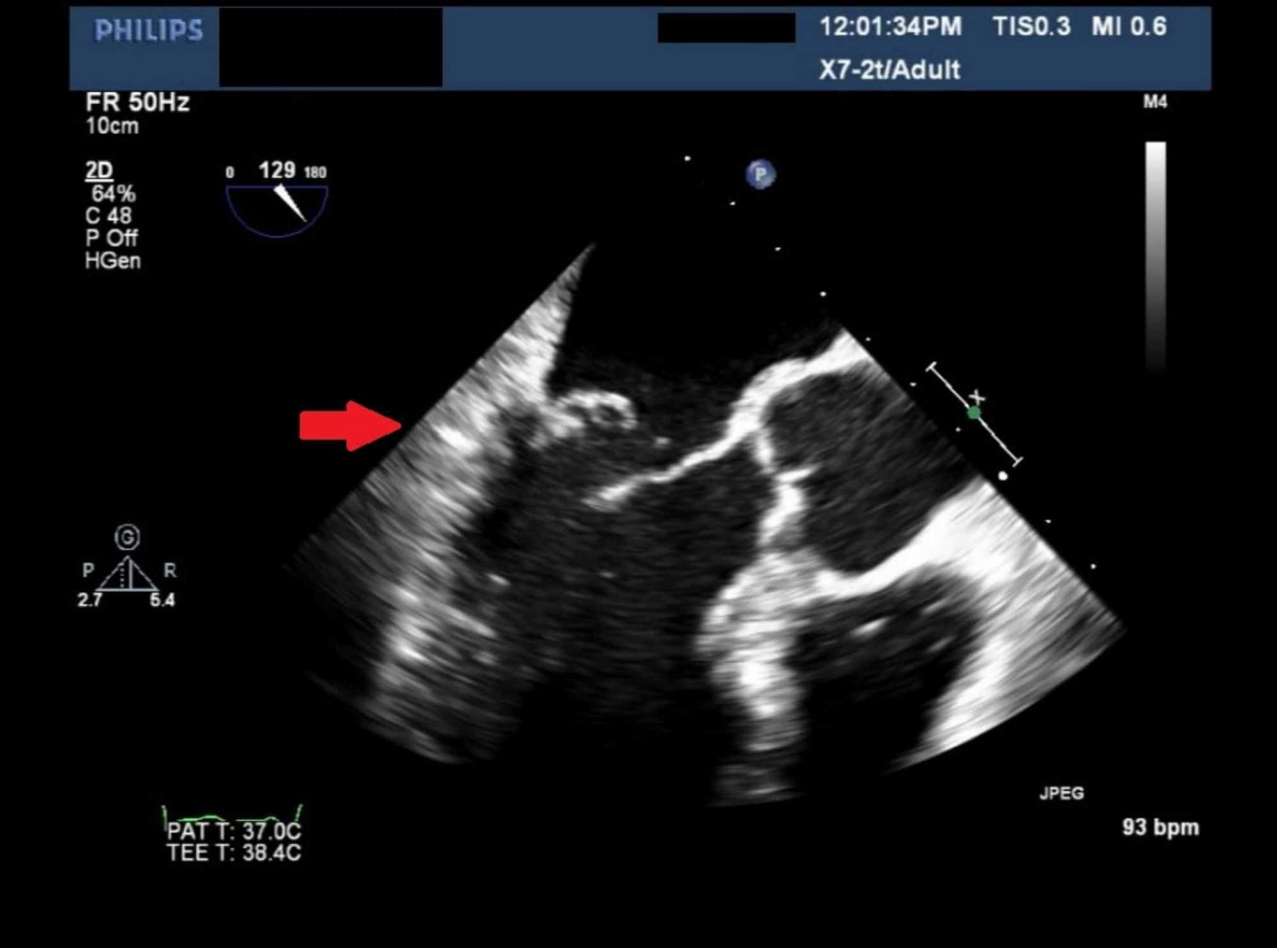
Two-dimensional echocardiography Anteriorly directed severe mitral regurgitation. Absent aortic valve disease.

**Figure 2:**
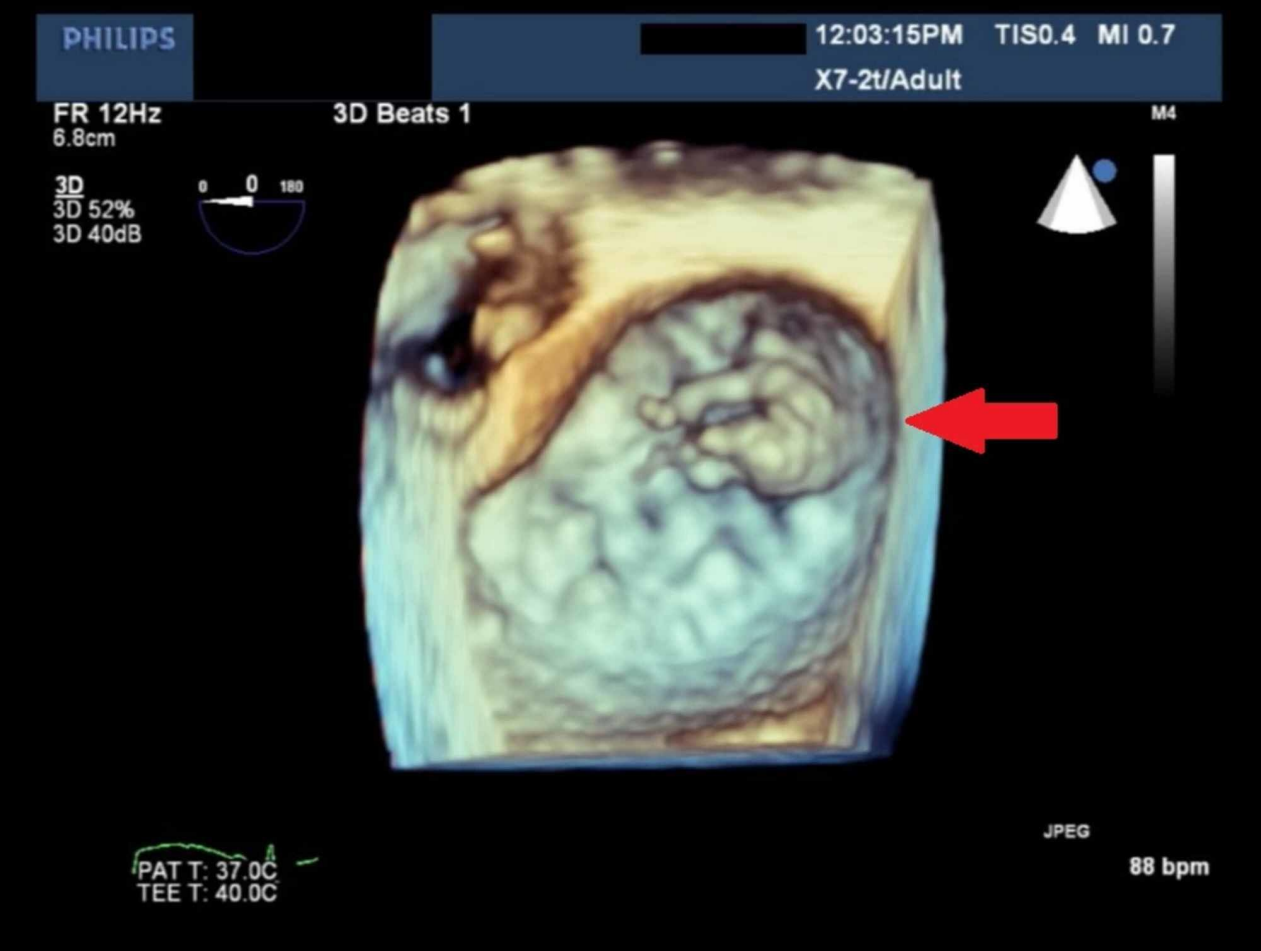
Transesophageal echocardiogram with 3D image acquisition Evidence of a flailed P3 scallop of the mitral valve leaflet. There is severe eccentric, anteriorly directed mitral regurgitation.

**Figure 3:**
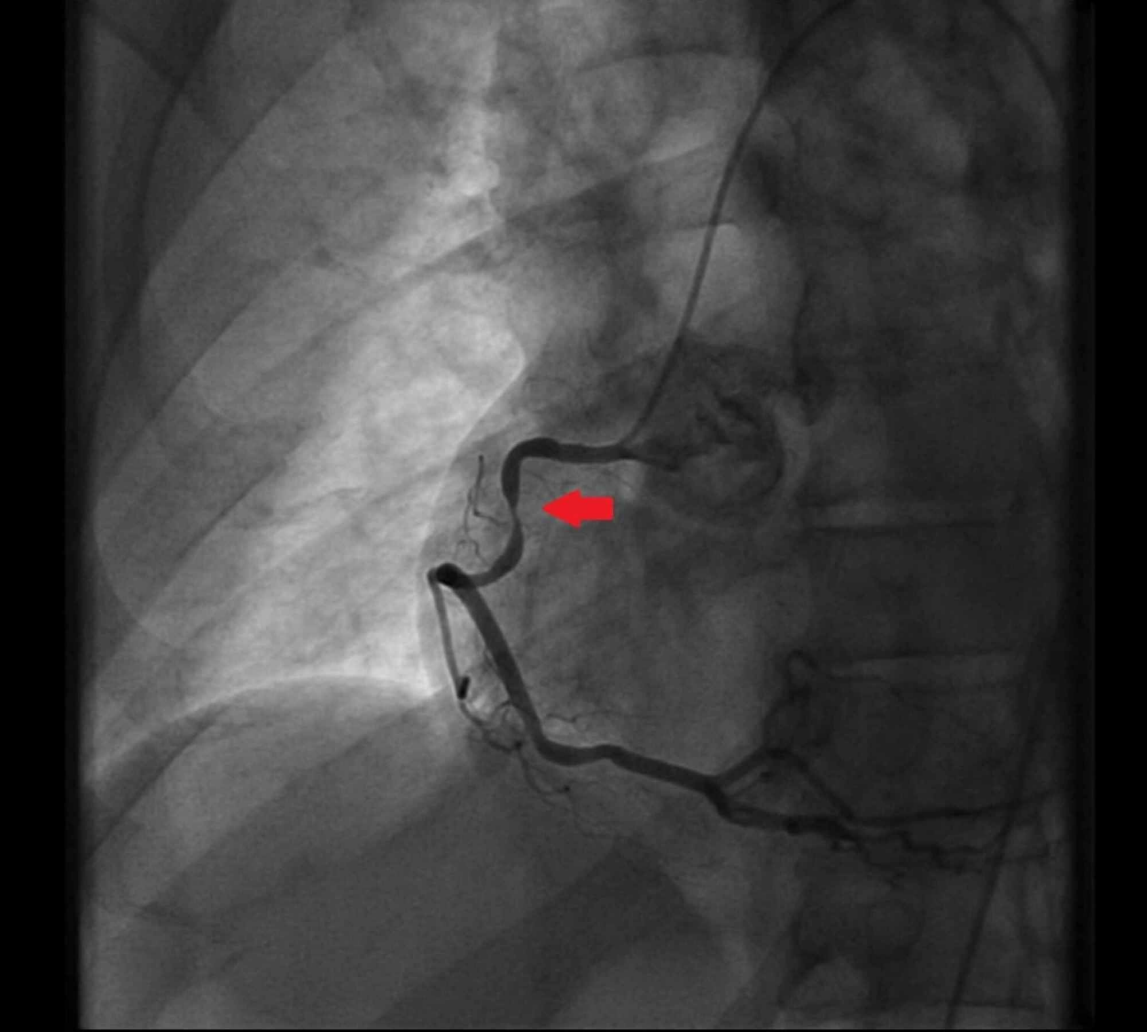
Coronary angiogram of the RCA The stenotic disease of the RCA. RCA: right coronary artery.

**Figure 4:**
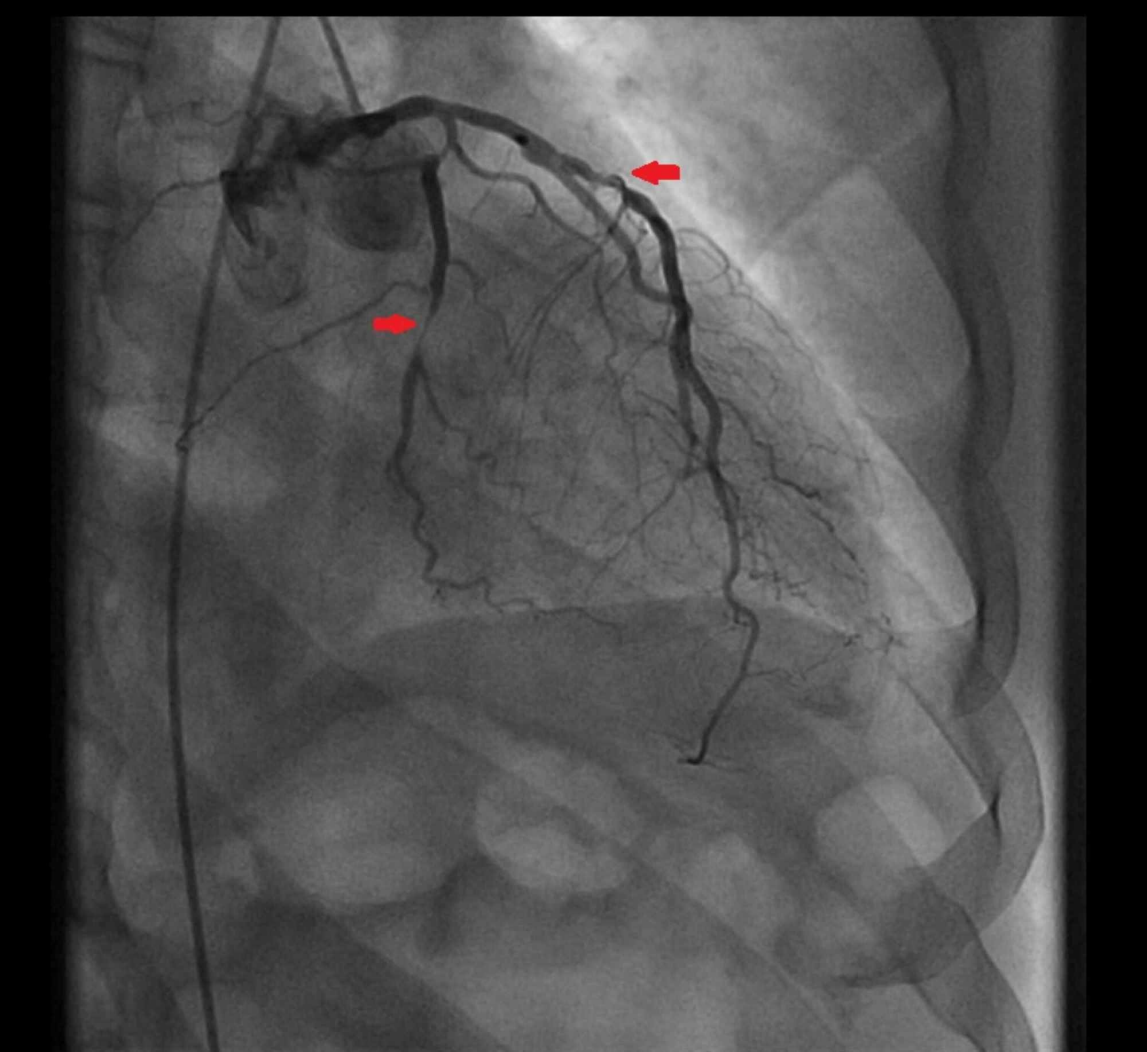
Coronary angiogram of the LAD artery The stenotic disease of the LAD and its septal branches. LDA: left anterior descending.

A 54-year-old male with a medical history of hypertension and transient ischemic attack, presented to the emergency department (ED) for evaluation of dyspnea on exertion. The patient reported that his dyspnea started one week prior to ED visit. He also endorsed orthopnea and intermittent chest pain. He also endorsed mild orthopnea and lightheadedness, though he denied any syncopal event. Vital signs were found within normal limits on arrival. He clinically appeared to be volume overloaded which improved quickly with IV furosemide. The physical examination also revealed a loud 3/6 systolic harsh murmur appreciated on the apex with radiation to the axillae. Laboratory testing was notable for an elevated troponin I of 0.10 ng/ml with a flat trend, elevated BNP of 301 pg/mL, lactate of 0.9 mmol/L, potassium of 3.8 mEq/L, sodium of 130 mEq/L, and magnesium of 2.1 mg/dl. The electrocardiogram showed sinus tachycardia with no ischemic changes and otherwise unremarkable. He underwent a 2D echocardiogram (Figure 1) with a subsequent transesophageal echocardiogram (TEE) with 3D image acquisition which was significant for hyper-dynamic LV function and evidence of isolated flailed P3 scallop of the MV leaflet resulting in a severe eccentric, anteriorly directed MR jet (Figure 2). The MV leaflets did not appear thickened and there was no evidence of mitral or aortic stenosis. Cardiac catheterization showed multivessel disease (Figures 3 and 4) for which the patient underwent coronary artery bypass grafting and MV repair.

## Discussion

This patient presented with new-onset congestive heart failure secondary to severe MR associated with undiagnosed MVP. The onset of the MR appeared to be relatively acute as his functional capacity was normal one week prior to his admission and lack of left atrial enlargement on imaging.

Typically, the middle scallop (P2) of the posterior leaflet is more prone to prolapse due to its redundancy and variable thickness with the impact of greater systolic pressure. However, in this case of acute severe MR, we identified an isolated flail of the P3 segment. Isolated P3 defect is rare; however, identifying the defect on TEE is paramount to provide an optimal surgical approach. Time spent to understand the dysfunction and to recognize lesions is critical for a successful operation. 

We believe that this rare TEE finding was associated with a torn chordae or ruptured papillary muscle secondary to ischemic disease. The posteromedial papillary muscle is typically affected by an inferior wall MI because its blood supply is solely from the posterior descending artery, usually a branch of the RCA, which was found to have severe stenotic disease on coronary angiogram in our patient. The anterolateral papillary muscle receives dual blood supply and is less commonly affected. This complication usually occurs during or within three or five days of non-ST-elevation or ST-elevation MI. In this case, the patient underwent successful coronary artery bypass grafting and mitral valve surgery.

A less likely explanation for his severe mitral regurgitation is the primary MVP. Primary MVP is characterized by myxomatous degeneration in the absence of any connective tissue pathology. In this case, the segmental mitral leaflet prolapse could possibly be secondary to myxomatous degeneration changes on the chordae tendinaeae, resulting in weakening and elongation. 

## Conclusions

TEE is paramount to identify the affected MV scallop segment, co-existing valve disease, assessment of severity, and planning MV surgery. Commonly, the middle scallop (P2) is more prone to prolapse due to its redundancy and variable thickness with the impact of greater systolic pressure. Interestingly, in this case of severe MR, we identified an isolated flail of the P3 segment. Isolated P3 defect is rare, however, identifying the defect on TEE is crucial to provide an optimal surgical approach. Time spent to understand the dysfunction and to recognize lesions is critical for a successful operation.
